# Box–Behnken Design-Based Optimization of Phytochemical Extraction from *Diplazium esculentum* (Retz.) Sw. Associated with Its Antioxidant and Anti-Alzheimer’s Properties

**DOI:** 10.3390/molecules29102204

**Published:** 2024-05-08

**Authors:** Sirawit Kongsung, Woorawee Inthachat, Boonrat Chantong, Uthaiwan Suttisansanee, Nattira On-Nom, Chaowanee Chupeerach, Sirinapa Thangsiri, Pornsiri Pitchakarn, Piya Temviriyanukul

**Affiliations:** 1 Master of Science Program in Toxicology and Nutrition for Food Safety, Institute of Nutrition, Mahidol University, Salaya, Phuttamonthon, Nakhon Pathom 73170, Thailand; sirawit.kon@student.mahidol.ac.th; 2 Institute of Nutrition, Mahidol University, Salaya, Phuttamonthon, Nakhon Pathom 73170, Thailand; woorawee.int@mahidol.ac.th (W.I.); uthaiwan.sut@mahidol.ac.th (U.S.); nattira.onn@mahidol.ac.th (N.O.-N.); chaowanee.chu@mahidol.ac.th (C.C.); sirinapa.tha@mahidol.ac.th (S.T.); 3 Department of Pre-Clinical and Applied Animal Science, Faculty of Veterinary Science, Mahidol University, Salaya, Phuttamonthon, Nakhon Pathom 73170, Thailand; boonrat.cha@mahidol.ac.th; 4 Department of Biochemistry, Faculty of Medicine, Chiang Mai University, Chiang Mai 50200, Thailand

**Keywords:** Alzheimer’s disease, antioxidant, BACE-1, *Diplazium esculentum*, edible ferns, human health, optimization, response surface methodology

## Abstract

A previous study reported that the ethanolic extract of the edible fern, *Diplazium esculentum* (Retz.) Sw. (DE), obtained from a non-optimized extraction condition exhibited anti-Alzheimer’s disease (AD) properties through the inhibition of a rate-limiting enzyme in amyloid peptide formation, β-secretase-1 (BACE-1). Nevertheless, a non-optimized or suboptimal extraction may lead to several issues, such as a reduction in extraction efficiency and increased time and plant materials. In this study, extraction of the DE was optimized to obtain appropriate BACE-1 inhibition using a Box–Behnken design (BBD) and response surface methodology (RSM). Data revealed that the optimal extraction condition was 70% (*v*/*v*) aqueous ethanol, 50 min extraction time, 30 °C extraction temperature, and 1:30 g/mL solid/liquid ratio, giving BACE-1 inhibition at 56.33%. In addition, the extract also exhibited significant antioxidant activities compared to the non-optimized extraction. Metabolomic phytochemical profiles and targeted phytochemical analyses showed that kaempferol, quercetin, and their derivatives as well as rosmarinic acid were abundant in the extract. The optimized DE extract also acted synergistically with donepezil, an AD drug suppressing BACE-1 activities. Data received from *Drosophila*-expressing human amyloid precursor proteins (APPs) and BACE-1, representing the amyloid hypothesis, showed that the optimized DE extract penetrated the fly brains, suppressed BACE-1 activities, and improved locomotor functions. The extract quenched the expression of glutathione S transferase D1 (GSTD1), inositol-requiring enzyme (IRE-1), and molecular chaperone-binding immunoglobulin (Bip), while donepezil suppressed these genes and other genes involved in antioxidant and endoplasmic reticulum (ER) stress response, including superoxide dismutase type 1 (SOD1), activating transcription factor 6 (ATF-6), and protein kinase R-like endoplasmic reticulum kinase (PERK). To sum up, the optimized extraction condition reduced extraction time while resulting in higher phytochemicals, antioxidants, and BACE-1 inhibitors.

## 1. Introduction

Alzheimer’s disease (AD) is a type of dementia that affects around 50 million people worldwide, and this number is expected to increase to 152 million people by 2050. The World Alzheimer’s Report 2019 stated that annual care costs for AD treatment among American people amounted to USD 340 billion [1], which could rise to USD 1.1 trillion by 2050. AD is an irreversible neurodegenerative disorder that causes a decline in cognitive abilities and impairs general behaviors [2]. The expected increase in AD patients in the near future will lead to significantly increased expenses for governments, communities, families, and people, as well as a decline in economic productivity [3]. The speedy development of AD medicines is urgently required and has attracted intensive research.

Several hypotheses have been posited regarding AD pathogenesis, including oxidative stress, impairment of the cholinergic nervous system, and the aggregation of amyloid (Aβ) peptides [4]. Naturally occurring amyloid peptides in the brain are generated through the proteolytic cleavage of amyloid precursor proteins (APPs) via two pathways. Non-toxic amyloid peptides are produced through non-amyloidogenic pathways, whereas toxic amyloid peptides, specifically Aβ_1–40_ and Aβ_1–42_, are formed via amyloidogenic pathways involving the cleavage of APPs by β-secretase-1 (BACE-1) and γ-secretase [5,6]. The accumulation of cytotoxic amyloid peptides leads to several adverse events in neurons, including amyloid plaques, oxidative stress, mitochondrial dysfunctions, neuroinflammation, endoplasmic reticulum (ER) stress, and eventually neuronal death [7,8,9]. Therefore, the reduction of oxidative stress and BACE-1 could be one approach to ameliorate the impact of AD. The US FDA has approved both lecanemab and aducanumab as novel immunotherapies to reduce amyloid plaques [10,11], highlighting the importance of the amyloid hypothesis in AD.

*Diplazium esculentum* (Retz.) Sw. (Pak-Kood in Thai), an edible fern, is a member of the Athyriaceae family, and is widespread across moist climatic areas, including the Philippines, India, China, and Thailand [12]. *D. esculentum* (DE) also contains non-nutritive compounds, including phytochemicals, and has been reported to promote health benefits such as treating skin diseases, asthma, and cancer as well as aiding in scar drying [13,14,15]. Previous studies demonstrated that the ethanolic extract of DE functioned as an anti-AD agent by quenching oxidative stress, inhibiting acetylcholine (AChE)-degrading enzymes (acetylcholinesterase (AChE) and butyrylcholinesterase (BChE)) in vitro, and inhibiting BACE-1 activities, resulting in the reduction of Aβ peptides in the brain and improved locomotor functions in *Drosophila* models of AD [16]. Interestingly, the ethanolic extract of DE also exhibited additive and synergistic effects with donepezil, which is an AD drug inhibiting both AChE and BACE-1 [17], to suppress BACE-1 [18].

These results implied that the ethanolic extract of DE obtained from a non-optimized extraction condition suppressed the development of AD by inhibiting BACE-1. Therefore, this study developed extraction conditions to achieve optimal BACE-1 inhibitory activities from *D. esculentum* using a Box–Behnken design (BBD) and response surface methodology (RSM). A BBD was utilized to examine the number of trials using a full factorial design that was both time-efficient and cost-effective, hence decreasing the probability of complications and simplifying the analysis while preserving a high level of precision, while RSM was employed to analyze the correlation between the studied factors under ideal circumstances using statistical and mathematical concepts [19]. Both BBD and RSM are widely used in the pharmaceutical and food industries to optimally recover bioactive phenolic and flavonoid compounds from various sources including plants for human health benefits while consuming less energy and raw materials [20]. This study investigated the effect of four ethanol extraction factors, including solvent concentration, extraction temperature, extraction time, and solid–liquid ratio (SLR), on BACE-1 inhibitory activities using BBD and RSM. The optimized DE extract showed significantly different antioxidant and BACE-1 inhibitory activities and required less extraction time compared to the non-optimized DE extract obtained from previous studies [16,18], affirming the advantages of BBD and RSM implementation in the optimization process. The optimized DE extract also impacted a number of genes linked to AD pathogenesis in a *Drosophila* model of the amyloid hypothesis, indicating a multi-target anti-AD agent function.

## 2. Results

### 2.1. Optimization of D. esculentum Extraction Conditions

#### 2.1.1. Model Fitting, Analysis of Variance, and Validation

We previously reported that the extraction of *D. esculentum* using ethanol as the solvent in a shaking water bath exhibited anti-AD properties, possibly via BACE-1 inhibition, as mentioned in the introduction [16]. Two non-optimized extraction conditions were previously reported, including (i) DE extracted with 70% ethanol at 30 °C for 2 h with the SLR at 1:10 [16], and (ii) DE extracted with 80% ethanol at 37 °C for 6 h with the SLR at 1:10 [18]. In this study, we aimed to improve the extraction conditions to obtain the optimal level of BACE-1 inhibition from *D. esculentum*.

Several factors contribute to the extraction efficiency of plant and herbal samples, such as raw materials, extraction methods, pressure, type of solvents, extraction time, extraction temperature, and material-to-solvent ratio [21]. To develop the extraction from previous studies [16,18], four extraction factors were selected for optimization, including ethanol concentration (X_1_), extraction time (X_2_), extraction temperature (X_3_), and solid–liquid ratio (SLR, X_4_). Twenty-seven BBD experiments or runs were performed (Table 8 and Table 9) to achieve optimal BACE-1 inhibition. Total phenolic content (TPC) was also optimized in this study because the bioactive phytochemicals in *D. esculentum* were hypothesized as phenolics [16]. The BACE-1 inhibitions ranged between 11.95% and 57.10% (Table 1), while TPC ranged between 6.63 and 16.40 mg GAE/g DW. Statistical analysis showed that varying extraction conditions resulted in different BACE-1 inhibition and TPC values, implying that the employed factors may influence both BACE-1 inhibition and TPCs.

**Figure 1:**
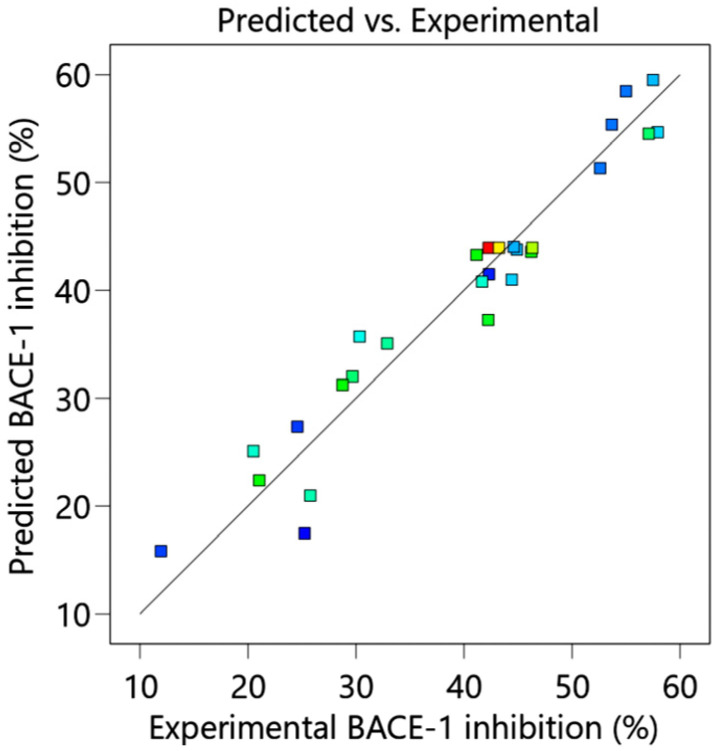
Experimentally measured BACE-1 inhibition vs. predicted value of *D. esculentum* calculated from Equation (1).

The data in Table 1 were confirmed by regression coefficients, *p* values of the second-order polynomial models for BACE-1 inhibition, and TPCs using one-way analysis of variance (ANOVA). The results in Table 2 show that only ethanol concentration played a significant role in optimizing BACE-1 inhibition of *D. esculentum* extract (*p* < 0.0001), while extraction time, temperature, and SLR had no effect on BACE-1 inhibition (*p* = 0.1233, 0.4886, and 0.1964, respectively). Model analysis for BACE-1 inhibition was also significant with a *p* value < 0.0001, while lack of fit was not significant, suggesting that the model was reliable. The R^2^ and R^2^ adjusted were 0.9353 and 0.8599 and close to 1.0, indicating high correlation between the predicted and experiment values (Figure 1). Pairwise interaction between the factors (X_1_X _2_, X_1_X_3_, X_1_X_4_, X_2_X_3_, X_2_X_4_, and X_3_X_4_) was insignificant, indicating no interaction between the indicated factors. In contrast, the *p* value for the model was greater than 0.5 for TPC (Table 2), and the R^2^ was 0.4923 (far from 1.0), implying that the model prediction of TPC was not valid; therefore, no predicted TPC values are shown in Table 1. In conclusion, Table 2 indicates that the model has high validity, it can be further used to predict the optimal extraction condition to obtain optimal BACE-1 inhibition from *D. esculentum*, and ethanol concentration was the only factor affecting this event, while it cannot be used to predict TPCs. The equation to obtain optimal BACE-1 was formulated as Equation (1).

(1) $$\begin{array}{l} \mathrm{BACE}-1\;\mathrm{inhibition}\;(\%)=-19.77+1.54X_{1}+0.10X_{2}-1.23X_{3}+1.63X_{4}-0.0026X_{1}X_{2}\\ +0.005X_{1}X_{3}+0.0007X_{1}X_{4}+0.0013X_{2}X_{3}+0.0092X_{2}X_{4}+0.015X_{3}X_{4}-0.0074X_{1}^{2}-0.0017X_{2}^{2}\\ +0.0041X_{3}^{2}-0.047X_{4}^{2}, \end{array}$$

where $X_{1}$, $X_{2}$, $X_{3}$, and $X_{4}$ represent independent values, including ethanol concentration (% *v*/*v*), time (min), temperature (°C), and SLR (g/mL).

#### 2.1.2. The Effect of Extraction Conditions on BACE-1 Inhibition

**Figure 2:**
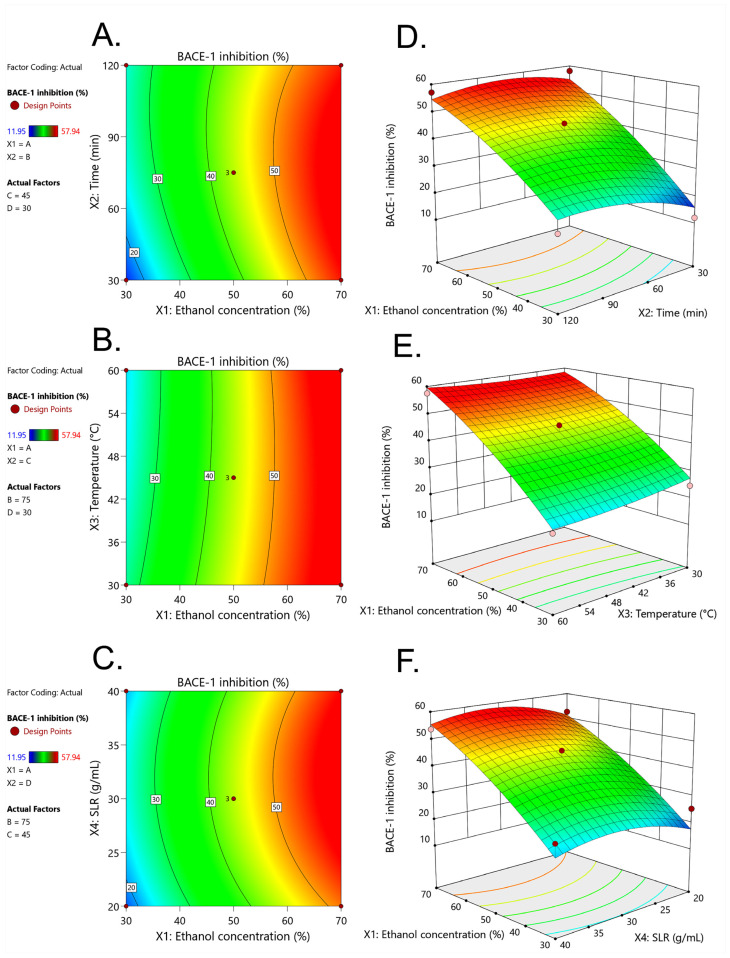

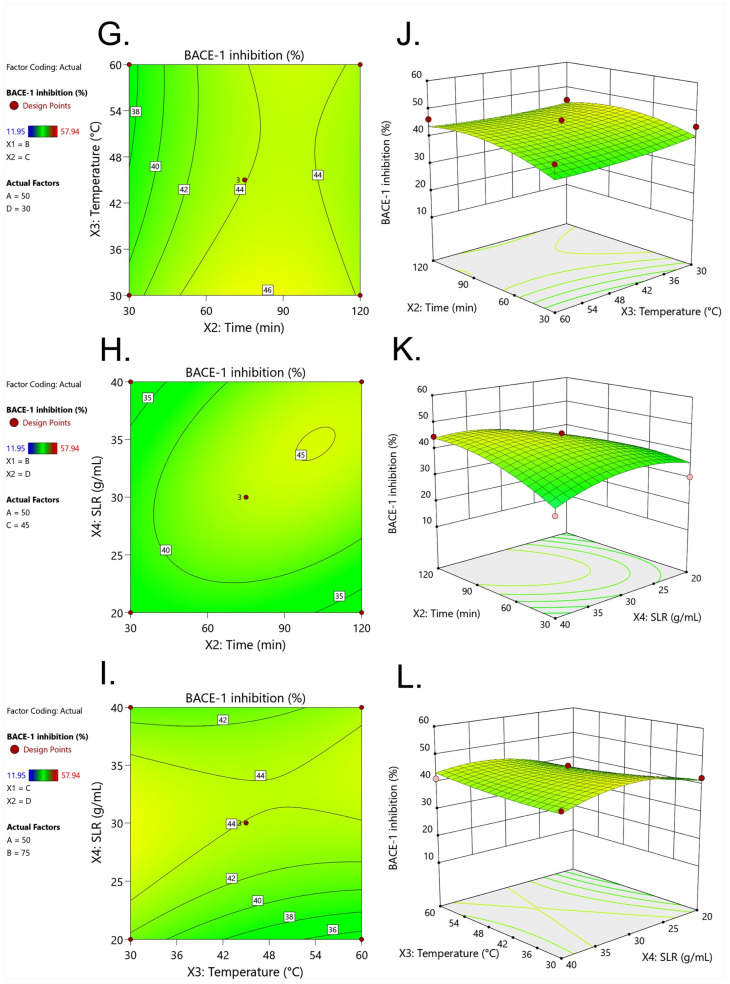
Contour plots (**A**,**C**,**E**) and response surface plots (**B**,**D**,**F**) of BACE-1 inhibition (%) affected by ethanol concentration (X_1_), extraction temperature (X_2_), extraction time (X_3_), and solid–liquid ratio, SLR (X_4_). Contour plots (**G**,**I**,**K**) and response surface plots (**H**,**J**,**L**) of BACE-1 inhibition (%) affected by ethanol concentration (X_1_), extraction temperature (X_2_), extraction time (X_3_), and solid–liquid ratio, SLR (X_4_).

The effects of ethanol concentration (X_1_), extraction time (X_2_), extraction temperature (X_3_), and SLR (X_4_) on obtaining the DE extract with optimal BACE-1 inhibition were further studied using RSM. The data were presented as contour and response surface plots (Figure 2). Figure 2A,D show contour and response surface plots of the interaction between ethanol concentration (X_1_) and extraction time (X_2_). The results revealed the main impact of ethanol concentration used for DE extraction on BACE-1 inhibition because increasing ethanol concentration (30% to 70%) but not extraction time resulted in increased BACE-1 inhibition. Similar results are shown in Figure 2C–F, indicating that BACE-1 inhibition increased only when ethanol concentration used for DE extraction was increased. Figure 2G–L show that no influence on BACE-1 inhibition was significantly observed when ethanol was not considered, confirming the ANOVA data in Table 2.

The optimized extraction conditions to acquire the highest BACE-1 inhibition from *D. esculentum* generated by Design-Expert software (version 13) were 69.8% (*v*/*v*) aqueous ethanol, 50 min extraction time, 30.4 °C extraction temperature, and 1:28.9 g/mL SLR, with a desirability value of 1.00. Under this condition, BACE-1 inhibition was 57.96%. For high reproducibility, we adjusted the condition to 70% (*v*/*v*) aqueous ethanol, 50 min extraction time, 30 °C extraction temperature, and 1:30 g/mL SLR and achieved BACE-1 inhibition at 56.33%, suggesting that the adjusted condition was reliable and could be used for subsequent experiments.

### 2.2. Metabolomic Profiling of Phytochemicals of Optimized D. esculentum Extract

Phytochemicals are bioactive compounds in plant extracts that possess several health benefits [22]. The metabolomic profiles of chemicals in the optimized *D. esculentum* extract were studied by HPLC-qTOF/MS using both positive and negative ion full scan modes. Agents with a library score (library matching score for fit) more than 90% are reported in Table 3 and Table 4. Table 3 displays fifty-five tentative compounds detected by the positive ion mode covering amino acids, vitamins, fatty acids, terpene derivatives, phytosterols, phenolics, flavonoids, and their derivatives. In this mode, according to the relative area, phytochemicals including rutin, quercetin, quercetin derivatives, and kaempferol glucosides were abundant. Table 4 lists twenty-nine tentative compounds detected by the negative ion mode, including amino acids, vitamins, fatty acids, phytosterols, saponins, phenolics, flavonoids, and their derivatives. Similar to the positive mode, rutin, rosmarinic acid, quercetin, quercetin derivatives, and kaempferol glucosides were abundant in the optimized DE extract.

### 2.3. Phytochemical Analysis of Optimized D. esculentum Extract using LC-ESI-MS/MS

Table 3 and Table 4 demonstrate abundant flavonoids in the optimized *D. esculentum* extract, including rutin, quercetin, quercetin derivatives, and kaempferol glucosides. However, HPLC-qTOF/MS could not be used as a quantitative analysis. Instead, we used a highly sensitive quantitative method (liquid chromatography–electrospray ionization–tandem mass spectrometry, LC-ESI-MS/MS) to determine the amounts of phytochemicals compared with twenty-six authentic standards. Table 5 displays the amounts of phytochemicals detected by LC-ESI-MS/MS. The findings revealed that kaempferol was the highest at 342.86 µg/g DW, followed by quercetin (less than kaempferol by 2.5-fold), fisetin, rosmarinic acid, and rutin. Previous research indicated that *D. esculentum* extract possessed high concentrations of kaempferol and quercetin, along with rutin and rosmarinic acid [16,18]. Fisetin, not previously reported in this plant, was also identified in the optimized extract. Therefore, based on both qualitative and quantitative analyses, kaempferol and quercetin were identified as the predominant flavonoids in *D. esculentum* extract.

### 2.4. Antioxidant Properties, BACE-1 Inhibition, and Synergistic Effect of Optimized DE Extract

As previously stated, DE extract obtained from a non-optimized extraction approach exhibited anti-AD properties by BACE-1 suppression (extraction with 70% ethanol at 30 °C for 2 h with SLR 1:10) [16]. Parameters including TPCs, total flavonoid content (TFC), antioxidant (DPPH radical scavenging, FRAP, and ORAC) activities, and BACE-1 inhibition obtained from both non-optimized and optimized extraction were compared because these parameters are crucial for the anti-AD properties of plant extracts and the data are shown in Table 6. Interestingly, all parameters derived from the optimized extraction were significantly different compared to the non-optimized extraction, indicating that the optimization of *D. esculentum* (DE) with BBD and RSM resulted in optimal DE extract for BACE-1 inhibition and also for phytochemicals and antioxidant activities.

One approach to treating AD is by developing novel drugs, while the study of the synergistic effects between AD drugs and phytochemicals may lead to improved therapeutic efficacy, reduce drug usage, and mitigate adverse effects. Thus, a combination of donepezil, which acts as both a cholinesterase and BACE-1 inhibitor [17], and AD drugs was studied. The results in Table 7 show that the optimized DE extract acted synergistically with donepezil to inhibit BACE-1, particularly the combination of IC_10_ DON + 0.25 mg/mL DE and IC_25_ DON + 0.125 mg/mL DE, which resulted in almost 50% BACE-1 inhibition. The optimized DE extract markedly enhanced the efficiency of DON as shown in the combination treatments of (i) IC_10_ DON ± 0.125 mg/mL DE, (ii) IC_10_ DON ± 0.25 mg/mL DE, and (iii) IC_25_ DON ± 0.125 mg/mL DE. This suggested that a reduced concentration of donepezil could suppress BACE-1 when combined with the optimized DE extract.

### 2.5. Anti-AD Properties of Optimized DE Extract in a Drosophila Model of AD

**Figure 3:**
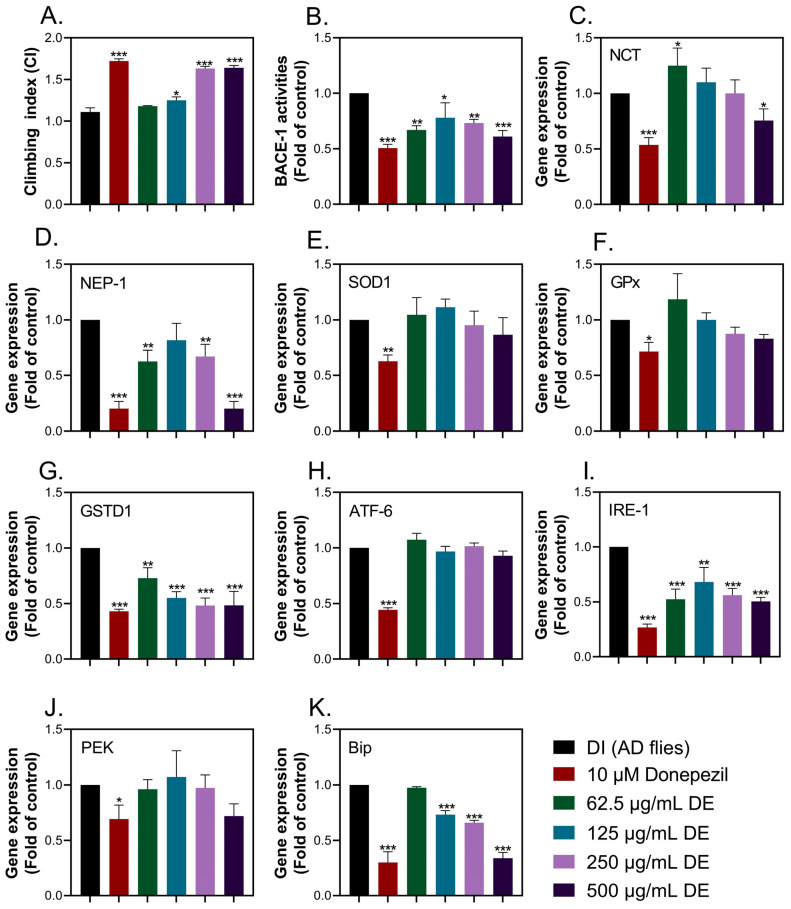
Anti-AD properties of DE extract in a *Drosophila* model of AD. (**A**). Climbing index on day 28 of treatment. (**B**). BACE-1 level on day 28 of treatment, (**C**). NCT expression on day 28 of treatment. (**D**). NEP-1 expression on day 28 of treatment. (**E**). SOD1 expression on day 28 of treatment. (**F**). GPx expression on day 28 of treatment. (**G**). GSTD1 expression on day 28 of treatment. (**H**). ATF-6 expression on day 28 of treatment and (**I**). IRE-1 expression on day 28 of treatment. (**J**). PEK or PERK expression on day 28 of treatment and (**K**). Bip expression on day 28 of treatment. One-day-old flies expressing human APP and BACE-1 were treated with DI, 10 µM donepezil, and optimized DE extract (62.5 to 500 µg/mL). At the indicated time, the flies were collected and tested for the climbing index, BACE-1 assay, or RT-qPCR. The values are mean ± SD of three independent experiments and statistical significance was analyzed against control (DI, black bar) by one-way ANOVA followed by Tukey’s multiple comparisons test. * *p* < 0.05, ** *p* < 0.01, and *** *p* < 0.001.

The optimized DE extract showed promising anti-AD results in vitro; however, one major obstacle for agents to treat neurodegenerative diseases is the ability to penetrate the blood–brain barrier (BBB). The ability of the optimized DE extract to cross the BBB was not proven by in vitro studies. Hence, we further tested the anti-AD properties using *Drosophila*-expressing human APPs and BACE-1 in fly brains, thus representing the amyloid hypothesis of AD. The AD flies were treated with DI (negative control), 10 µM donepezil (AD drug), and optimized DE extract (62.5 to 500 µg/mL) for 28 days. After that, the flies were tested for climbing index and then the fly heads were determined for BACE-1 activities and the expression of various genes involved in AD, as shown in Figure 3. Firstly, the flies were assayed for locomotor function by the climbing assay. Data revealed that AD flies (DI) showed a low climbing index (CI), while CI increased in donepezil-treated flies, inferring that donepezil could rescue the locomotor function of AD flies. The same results were seen in flies exposed to DE extract (125 to 500 µg/mL) but not for 62.5 µg/mL (Figure 3A). A similar pattern was found in the BACE-1 assay, which is a rate-limiting step in amyloid peptide formation [23]. AD flies with DI exhibited high levels of BACE-1 activities, which significantly reduced when the flies were treated with donepezil and DE extract (125 to 500 µg/mL) (Figure 3B).

Genes involved in AD pathogenesis, including proteins in the amyloid pathway such as nicastrin (NCT) and neprilysin 1 (NEP-1); antioxidant proteins such as superoxide dismutase type 1 (SOD1), glutathione peroxidase (GPx), and glutathione S transferase D1 (GSTD1); and proteins in endoplasmic reticulum (ER) stress such as activating transcription factor 6 (ATF-6), inositol-requiring enzyme (IRE-1), protein kinase R-like endoplasmic reticulum kinase (PERK or PEK), and molecular chaperone-binding immunoglobulin (Bip), were studied for their expression using RT-qPCR.

NCT is a protein complex with γ-secretase, another enzyme that cuts peptides from BACE-1 [24]. Figure 3C shows that NCT expression declined in donepezil-treated flies with a high dose of DE extract (500 µg/mL). NEP-1 is an amyloid-degrading enzyme [25]. NEP-1 expression was reduced by approximately 5-fold in donepezil-exposed flies, implying that fewer amyloid peptides were formed in these flies. Gradually increasing the DE concentration also reduced NEP-1 expression, especially at the highest dose (Figure 3D).

Amyloid peptides are neurotoxic and induce oxidative stress and neuronal death [26,27,28]. Figure 3F–H demonstrate that the optimized DE extract was not able to suppress the expression of antioxidant proteins such as SOD1 and GPx, except for GSTD1, compared to donepezil in AD flies, indicating that DE extract and donepezil exhibited anti-AD properties but may follow different antioxidant mechanisms.

ER stress activation relies on three transducer proteins, such as ATF-6, IRE-1, and PEK or PERK. In the normal condition, Bip binds to these three transducers and suppresses the ER stress response [29]. Misfolded proteins or protein aggregation including amyloid peptides trigger the release of Bip from transducer proteins, leading to ER stress responses. Figure 3H–K illustrate that donepezil significantly suppressed the ER stress response by decreasing the expression of ATF-6, IRE-1, PEK, and Bip. Interestingly, the DE extract was only capable of inhibiting IRE-1, while the DE extract, even at the highest dose, could not reduce ATF-6 and PEK expression, implying that Bip expression reduced by DE extract was solely dependent on IRE-1. Similar to the expression of antioxidant proteins, the results suggested that although both donepezil and the optimized DE extract acted as anti-AD agents, they functioned using different mechanisms, particularly in the regulation of oxidative stress and ER stress response.

## 3. Discussion

Alzheimer’s disease (AD), a type of dementia, is projected to become the primary cause of global mortality [1]; therefore, comprehensive studies are investigating novel pharmaceuticals to treat AD. Therapeutic agents, including cholinesterase (AChE and BChE) and BACE-1 inhibitors as well as antioxidants, are among those undergoing investigation for AD treatment. Previous reports revealed that *Diplazium esculentum* (DE) extract suppressed the key enzymes involved in AD pathogenesis, including AChE, BChE, and BACE-1. Particularly, the extract inhibited BACE-1 activities and subsequently decreased amyloid peptides in the brain of *Drosophila* expressing APPs and BACE-1 (a model for the amyloid pathway) [16]. The DE extract utilized in the previous study was derived under suboptimal extraction conditions and the optimal BACE-1 inhibition from the DE extract remains unclear. Non-optimized extraction may lead to several issues, such as reductions in extraction efficiency and increased time and plant materials. In this study, the extraction conditions were optimized to achieve the appropriate BACE-1 inhibitor from DE, with BBD and RSM employed in the experimental design and data interpretation. The optimized DE extract was then subjected to phytochemical profile analysis, antioxidant activities, BACE-1 inhibitory activities, and anti-AD activities in the *Drosophila* model of the amyloid pathway.

The optimized extraction condition for maximal BACE-1 inhibition was 70% (*v*/*v*) aqueous ethanol, 50 min extraction time, 30 °C extraction temperature, and 1:30 g/mL SLR. Under this condition, 56.33% of BACE-1 inhibition was observed. The optimized extraction condition was quite similar to the non-optimized extraction [16], which was 70% (*v*/*v*) aqueous ethanol, 120 min extraction time, 30 °C extraction temperature, and 1:10 g/mL SLR [16], but the optimized condition resulted in a reduction of 70 min of extraction time. In addition, compared to Inthachat et al. (2024) [18], the optimized condition consumed less extraction time by almost 300 min. Compared to the non-optimized extraction, the optimized extraction condition also yielded significantly higher levels of TPCs, TFCs, BACE-1 inhibitory activities, and antioxidant activities (DPPH, FRAP, and ORAC) (Table 6). The results suggested that the optimized extraction condition increased phytochemical contents and bioactivities while reducing the extraction time. Four extraction factors, including ethanol concentration, extraction time, extraction temperature, and SLR, were studied. The data revealed that none of these factors were associated with TPCs. Ethanol concentration was significantly correlated with BACE-1 inhibition, with an increasing ethanol concentration resulting in higher BACE-1 inhibition (Figure 2), implying that BACE-1 inhibitors from DE may have a low polarity index (PI) since 30% aqueous ethanol has a PI higher than 70% ethanol [30]. Likewise, BACE-1 inhibition did not decrease at an extraction temperature of 60 °C based on the absence of a decrease in BACE-1 inhibition at an extraction temperature of 60 °C. The data suggested that the BACE-1 inhibitors present in the DE extract were heat-stable (Figure 2D). Interestingly, the interaction between ethanol concentration and the other extraction factors was insignificant, suggesting the importance of ethanol as a single factor to acquire optimal BACE-1 inhibition. Surprisingly, none of the four extraction factors contributed to TPCs. Previous studies produced contradictory results. Azahar et al. (2017) [31] reported that extraction time, SLR, and temperature contributed to TPCs from *Curcuma zedoaria* leaves, while Zhang et al. (2021) [32] also found that all four extraction factors contributed to TPCs of *Empetrum nigrum* fruits. Compared with other organic solvents, ethanol is commonly used in food and nutraceutical sectors because of its safety and edibility [33], with aqueous ethanol the most appropriate solvent to extract phenolics from plant samples [34]. Because soluble phenolics are predominantly accumulated in the epidermal and sub-epidermal (outer layers) [35], we hypothesized that a low concentration of ethanol (30%) might be strong enough to recover most of the phenolics from DE, an edible fern with a soft and thin wall. Thus, increasing the ethanol concentration to 70%, even in combination with other extraction factors, may not increase the TPC since it may have already reached saturation.

The optimized DE extract was subjected to metabolomic phytochemical analysis to detect the abundance of tentative compounds. To the best of our knowledge, this is the first study investigating the metabolomic phytochemical profiles of DE. Phytosterols, phenolics, flavonoids, amino acids, and fatty acids comprised the majority of the identified compounds in DE extract, in accordance with previous reports [36]. For phytochemicals, the DE extract contained rosmarinic acid, kaempferol glucosides, quercetin, quercetin derivatives, rutin, ferulic acid, piperine, capsaicin, campesterol, and mangostin. The targeted LC-ESI-MS/MS confirmed kaempferol, quercetin, rutin, and rosmarinic acid. Our previous study also identified kaempferol, quercetin, rosmarinic acid, and rutin in DE samples collected in the same area but in a different collecting year [18]. Unfortunately, the metabolic study failed to detect morin, cinnamic acid, eriodictyol, 5-O-methyl ether, 7-O-β-D-xylosylgalactoside, syringic acid, protocatechuic acid, myricetin, and three ecdysteroids (amarasterone A1, makisterone C, and ponasterone A), as formerly documented [37,38,39,40], possibly due to different planting areas and extraction methods.

Synergistic use with approved medications is an alternative way of treating ailments. Phytochemicals are the subject of extensive study for this approach due to their low cost and safety. An ethanolic extract of propolis combined with donepezil improved memory better than monotherapy [41], while the combination of gallic acid with donepezil synergistically inhibited AChE activities in the brain of aluminum chloride-induced neurotoxicity in rats [42]. In this study, we found that the optimized DE extract acted synergistically with donepezil to impede BACE-1 activities, especially when low doses of donepezil and DE extract were combined, suggesting that low doses of donepezil can be used in combination with DE extract to attain optimal BACE-1 inhibition (Table 7). Quercetin may also inhibit BACE-1 when combined with donepezil in a synergistic manner, whereas kaempferol may have a slight antagonistic effect with BACE-1 [18]. Regarding BACE-1 inhibition, molecular docking showed that donepezil interacts with the active site of BACE-1 with a half-maximal inhibitory concentration (IC_50_) of 1.5 µM [17], while an in vitro study and molecular docking demonstrated that quercetin and kaempferol bind directly to the active site of BACE-1, albeit with different binding capacity, leading to IC_50_ values of 5.4 µM and 14.7 µM, respectively. A slight antagonistic effect of kaempferol against donepezil may result from competition for the active site of BACE-1 or from kaempferol binding, causing a minor conformational change in BACE-1, which hinders donepezil binding. In contrast, the inhibitory effects of BACE-1 and the reduction in amyloid peptides Aβ_1-40_ and Aβ_1-42_ in neuronal cells were observed exclusively with quercetin [43], suggesting that while kaempferol was abundant in the optimized DE extract, quercetin may possess bioactive anti-AD properties in vivo. No experimental data were available for fisetin and BACE-1 but the molecular docking study showed that fisetin can bind to the active site of BACE-1 with a significant ligand-binding complex [44]. Rosmarinic acid, a polyphenol compound, showed non-competitively inhibited BACE-1 activities with IC_50_ at 21 µM, suggesting that rosmarinic acid interacted with BACE-1 in other areas but not at the enzyme active site [45]. Thus, rosmarinic acid might function together with quercetin to inhibit BACE-1. In summary, with the possible exception of kaempferol, three compounds (quercetin, fisetin, and rosmarinic acid) might contribute to BACE-1 inhibition in the optimized DE extract.

The cleavage of APPs by BACE-1 leads to the formation of amyloid peptides, which are eventually aggregated and accumulated. Aggregated amyloid peptides are neurotoxic and are capable of activating various cellular pathways, including oxidative stress and ER stress responses [27,28]. Thus, to elucidate the anti-AD properties of optimized DE extract, we employed *Drosophila*-expressing human APPs and BACE-1, which represent the amyloid hypothesis. The optimized DE extract (125–500 µg/mL) reduced BACE-1 activities, leading to improvement in the locomotor function. Notably, donepezil suppressed all examined genes implicated in amyloid degradation, antioxidant enzymes, and the ER stress response, while the optimized DE extract only affected NEP-1, GSTD1, IRE-1, and Bip. Neprilysin (NEP-1) plays a crucial role in the degradation of amyloid peptides, thereby mitigating their buildup and the resulting neuronal damage linked to AD [46]. Nevertheless, in this study, the mRNA expression of NEP-1 exhibited a decrease in flies treated with optimized DE extract. This decline in NEP-1 expression was attributed to reduced levels of aggregated amyloid peptides in fly heads, implying that diminished enzyme activity is required for the clearance of aggregated amyloid peptides. Glutathione S-transferases (GSTs) are a group of enzymes involved in the detoxification of electrophilic compounds through conjugation with glutathione, which consists of three amino acids (glycine, cysteine, and glutamic acid). Although not directly involved in amyloid peptide metabolism, GSTD1 plays a cellular defense role against oxidative stress and neuroinflammation and indirectly affects amyloid peptide levels. Elevated oxidative stress in AD flies may trigger GSTD1 upregulation to counteract damages [46,47]. The optimized DE extract decreased GSTD1 expression in this study, due to reduced aggregated amyloid peptide-stimulated oxidative stress. In AD, misfolded proteins like amyloid peptides accumulate in the ER of neurons, inducing ER stress and activating the unfolded protein response (UPR), including the IRE-1 signaling pathway. IRE-1 activation in AD may exacerbate neuroinflammation, synaptic dysfunction, and neuronal cell death. Bip, an ER chaperone, serves as a primary sensor for unfolded proteins. Dysregulation of Bip expression and function is implicated in AD. IRE-1 signaling and Bip dysregulation contribute to AD pathogenesis by promoting neuronal dysfunction, neuroinflammation, and neuronal death [48,49,50]. Notably, a recent study demonstrated that an optimized DE extract reduced IRE-1 and Bip expression, suggesting a potential role in mitigating ER stress and decreasing AD pathogenesis. In summary, the optimized DE extract repressed not only BACE-1 activities in AD flies but also the downstream response of aggregated amyloid peptides, including oxidative stress and ER stress response.

## 4. Materials and Methods

### 4.1. Sample Preparation

Leaves of *Diplazium esculentum* (Retz.) Sw. (DE) were collected from Chiang Mai, Thailand. The samples were deposited at the Bangkok Herbarium (BK), Bangkok, Thailand (voucher specimens: BK069943). Young fronds were cleaned twice with deionized water (DI), cut into pieces, and air-dried for 2 h at room temperature. Then, the samples were freeze-dried by freeze-dryer (Powerdry PL9000 from Heto Lab Equipment (AllerØd, Denmark)) and ground into fine powder. The moisture content was then measured using a moisture analyzer (HE53 series, Mettler-Toledo AG, Greifensee, Switzerland). The sample was kept at −20 °C until used.

### 4.2. Sample Extraction

Four factors, including ethanol concentration (30–70%, diluted with type I water) (X_1_), extraction time (30–120 min) (X_2_), extraction temperature (30–60 °C) (X_3_), and solid–liquid ratio (SLR) (1:20–1:40) (X_4_), were subjected to the Box–Behnken design (BBD). The BBD matrix with independent variables is shown in Table 8. After analysis, BBD resulted in a total 27 of extraction conditions as shown in Table 9.

**Table 8:** Box–Behnken design matrix with independent variables and their actual levels.

Factors	Unit	Actual Levels		
		−1	0	1
(X_1_) Ethanol concentration	(% *v*/*v*)	30	50	70
(X_2_) Extraction time	min	30	75	120
(X_3_) Extraction temperature	°C	30	45	60
(X_4_) Solid–liquid ratio (SLR)	*w*/*v*	20	30	40

**Table 9:** Uncoded BBD of four independent variables (X_1_ to X_4_) derived from Table 8.

Run	X_1_: Ethanol (% *v*/*v*)	X_2_: Time (min)	X_3_: Temperature (°C)	X_4_: SLR (g/mL)
	Uncoded	Uncoded	Uncoded	Uncoded
1	50	75	60	20
2	30	75	45	20
3	70	75	30	30
4	50	30	30	30
5	30	75	60	30
6	50	30	60	30
7	50	75	45	30
8	70	75	45	20
9	50	120	30	30
10	70	75	60	30
11	30	75	45	40
12	70	30	45	30
13	50	120	45	20
14	50	30	45	40
15	30	75	30	30
16	30	120	45	30
17	50	75	30	20
18	50	75	60	40
19	50	120	60	30
20	70	75	45	40
21	50	75	30	40
22	50	75	45	30
23	70	120	45	30
24	50	30	45	20
25	30	30	45	30
26	50	120	45	40
27	50	75	45	30

The powdery sample was extracted based on Table 9 using a water bath shaker (Shaking water bath, SWB-35, HYSC, Seoul, Republic of Korea). Then, supernatants were centrifuged at 4500× *g* at 4 °C for 20 min (Centrifuge 5430R, Eppendorf, Hamburg, Germany). The supernatant was evaporated to remove ethanol using an evaporator (N-1200B series rotary evaporator with OSB-2100 bath, EYELA, Bohemia, NY, USA). The dry extract was re-dissolved in dimethyl sulfoxide (DMSO) prior to further analysis.

### 4.3. Phytochemical Analysis

#### 4.3.1. Total Phenolic Contents (TPCs) and Total Flavonoid Contents (TFCs)

TPCs were measured using Folin–Ciocalteu reagent with the well-established protocol [51]. The TPCs were assayed at 765 nm using a microplate reader (SpectraMax^®^ M5, San Jose, CA, USA). Gallic acid (10–200 µg/mL) was used as a standard compound and the data were reported in terms of milligrams of gallic acid equivalent per gram of dry weight (mg GAE/g DW)

TFCs were measured using aluminum chloride solution with the well-established protocol reported in [52]. The TFCs were evaluated at 510 nm using a microplate reader (SpectraMax^®^ M5, San Jose, CA, USA). Quercetin (10–250 µg/mL) was used as a standard, and the data were reported in terms of milligrams of quercetin equivalent per gram of dry weight (mg QE/g DW).

#### 4.3.2. Metabolomic Profiling (Non-Targeted) of Phytochemicals Using High-Performance Liquid Chromatography (HPLC)–Tandem Quadrupole Mass Spectrometer (HPLC-qTOF-MS)

The optimized extract of *D. esculentum* was re-dissolved in dimethyl sulfoxide (DMSO) and filtered through a 0.2 µm nylon filter. Prior to injection, the extract was diluted with type I water to a final concentration of DMSO at 0.1%. A 2 µL solution (without acid hydrolysis) was loaded on an AcclaimTM RSLC 120 C18 column (100 mm × 2.1 mm, 2.2 µm particle size, pore size 120 Å, Thermo Fisher Scientific, Waltham, MA, USA), which was connected to HPLC-qTOF-MS (TripleTOF^®^ 6600 ± quadrupole time-of-flight mass analyzer, AB SciEx, Framingham, MA, USA). A gradient mobile phase consisting of 0.1% formic acid in water (solvent A) and 0.1% formic acid in acetonitrile (solvent B) with a 0.4 mL/min flow rate for 30 min was used for the separation as follows: 0.00–1.00 min, 1–1% B; 1.00–21.00 min, 1–80% B; 21.00–21.10 min, 80–95% B; 21.10–25.00 min, 95–95% B; 25.00–25.10 min, 95–1% B; 25.10–30.00 min, 1–1% B. Molecular mass analysis was evaluated in both positive ion and negative ion full scan mode with a mass range of 100–1000 *m*/*z*. To identify potential chemicals, the MS spectra were aligned with a database of the natural products high-resolution MS/MS Spectral Library on SCIEX OS (AB SciEx, Framingham, MA, USA) and the National Institute of Standards and Technology (NIST). Tentative compounds with a possibility score (library score) of more than 90% were reported.

#### 4.3.3. Targeted Phytochemical Profile Analysis Using Liquid Chromatography–Electrospray Ionization–Tandem Mass Spectrometry (LC-ESI-MS/MS)

The dried extract was re-dissolved under the acid hydrolysis method as previously reported [51]. In this condition, the glycosidic bond between sugar and phenolics is disrupted, leading to its aglycone. Briefly, the extract was re-dissolved in formic acid and 62.5% (*v*/*v*) methanol containing *tert*-butyl hydroquinone, then shaken at 80 °C for 2 h and filtered through a 0.22 µm polytetrafluoroethylene (PTFE) filter. The extract was then loaded to an Accucore RP-MS column (a 2.1 mm × 100 mm, 2.6 μm column (Thermo Fisher Scientific, Bremen, Germany), which was connected to the LC–ESI–MS/MS system consisting of a Dionex Ultimate 3000 series ultra-high-performance liquid chromatograph (UHPLC) system, a diode array detector, a TSQ Quantis Triple Quadrupole mass spectrometer (MS), and a Chromeleon 7 chromatography data system (version 7.2.9.11323, Thermo Fisher Scientific, Bremen, Germany).

Acetonitrile (A) and 0.1% *v*/*v* formic acid in Milli-Q water (B) were used as mobile phase. A gradient solvent system was set as follows: 0.0–0.8 min, 10–80% A; 8.0–8.1 min, 80–10% A; 8.1–10.0 min, 10% A, at a flow rate of 0.5 mL/min. Supplementary Tables S1 and S2 show a list of authentic standards with parameters and validations, respectively.

### 4.4. Determination of Antioxidant Activities

2,2-diphenyl-1-picryhydrazyl (DPPH) radical scavenging, ferric reducing antioxidant power (FRAP), and oxygen radical antioxidant activity (ORAC) assays were performed to evaluate the antioxidant abilities of the optimized DE extract. All spectrophotometric assays were performed using well-developed protocols as previously detailed without modification [52]. The results were expressed as µmol of Trolox equivalent per gram of dry weight (µmol of TE/g DW).

### 4.5. Determination of BACE-1 Inhibitory Activities

BACE-1 activities were tested using a BACE-1 activity assay kit (Sigma-Aldrich, St. Louis, MO, USA) following the manufacturer’s instructions. The fluorescence signal amplification is detected subsequent to substrate cleavage by BACE-1 in the assay, which is founded upon the fluorescence resonance energy transfer (FRET) method.

### 4.6. Determination of Synergistic Effects of the Optimized D. esculentum (DE) Extract

Tests on the synergistic effects between the optimized D. esculentum extract and donepezil (AD drug) were performed as formerly detailed [53,54]. In brief, DE extract, donepezil, and a mixture of DE extract and donepezil were assayed for BACE-1 inhibition. The synergistic effects were calculated by using the theoretical value (TV) and the real experiment value (EV) as in Equation (2), when the enhancing effect was observed:

(2) $$\mathrm{TV}=\left(\frac{\mathrm{EV}\;\mathrm{of}\;\mathrm{donepezil}}{2}\right)+\left(\frac{\mathrm{EV}\;\mathrm{of}\;\mathrm{DE}\;\mathrm{extract}}{2}\right)$$

Interpretation is as follows: synergistic effect when the TV is more than 5% (>5%) below EV, antagonistic effect when the TV is more than 5% above EV, and additive effect when TV and EV differ by <5%.

### 4.7. Drosophila Stocks, Culture, and Treatment

Flies, including elav-GAL4 and UAS-APPs-BACE-1 (BDSC 33798), were procured from Bloomington *Drosophila* stock center, Indiana University, USA. All flies were cultured and maintained in a standard medium at 25 °C, 60% humidity, and a 12 h/12 h light–dark cycle in climate chambers with ICH-compliant light sources (BINDER GmbH, Tuttlingen, Germany). Mating between them resulted in F1 progenies expressing human amyloid precursor proteins (APPs) and β-secretase-1 (BACE-1), particularly in the fly brains. Newly elosed F1 flies were treated with the optimized DE extract (62.5 to 500 µg/mL) and donepezil (10 µM) for 28 days and the food was replaced every three days.

### 4.8. Locomotor Assay or Climbing Assay

Locomotor assay or climbing or assay was determined as previously described [55]. Briefly, on the indicated day after treatment, flies were relocated to a clean glass tube without anesthesia and given 15 min for acclimatization at 25 °C. Flies were tapped to bring them to the bottom and then we allowed them to climb for 5 s. The climbing index (CI) was calculated as in Equation (3):

(3) $$\mathrm{CI}=\frac{\mathrm{the}\;\mathrm{number}\;\mathrm{of}\;\mathrm{flies}\;\mathrm{in}\;\mathrm{each}\;\mathrm{score}\;\times \;\mathrm{the}\;\mathrm{score}\;\mathrm{they}\;\mathrm{reached}}{\mathrm{the}\;\mathrm{total}\;\mathrm{number}\;\mathrm{of}\;\mathrm{flies}\;\mathrm{in}\;\mathrm{each}\;\mathrm{group}}$$

### 4.9. Determination of Gene Expression in Drosophila Using Reverse Transcription-Quantitative Polymerase Chain Reaction (RT-qPCR)

Fly heads were collected on day 28 of treatment and extracted for RNA using TRIZol reagent (Invitrogen, Waltham, MA, USA). The cDNA was generated using ReverTra Ace™ qPCR RT Master Mix with gDNA Remover (Toyobo, Osaka, Japan) and the PCR reaction was assayed using THUNDERBIRD™ SYBR qPCR Mix (Toyobo, Osaka, Japan) with the PCR conditions as follows: denaturation at 95 °C for 10 min, followed by 40 cycles of denaturation at 95 °C for 30 s, annealing at 60 °C for 30 s, and elongation at 72 °C for 30 s. All primers, except a housekeeping gene (RpL32) [55], were designed using the NCBI primer designing tool. The list and sequences of primers are provided in Supplementary Table S3. The expression level of each gene was normalized to RpL32 and then the fold change compared to the control was reported.

### 4.10. Statistical Analysis

BBD and RSM were performed using Design–Expert version 13 (Stat-Ease, Inc., Minneapolis, MN, USA). All experiments were assayed in triplicate (*n* = 3) and presented as mean ± standard deviation (SD). The one-way analysis of variance (ANOVA) and Duncan’s multiple comparisons, Tukey’s multiple comparisons test, or an unpaired t-test (as indicated in the table legend) were performed using SPSS version 18 (Statistical Package for the Social Sciences, SPSS Inc., Chicago, IL, USA) to test the difference between samples. *p* ≤ 0.05 was considered a significant difference.

## 5. Conclusions

Extraction conditions were optimized to attain optimal BACE-1 inhibitors from the edible fern, *Diplazium esculentum* (DE). The optimized extraction condition was quite similar to the non-optimized condition but required considerably less extraction time while resulting in significant phytochemicals, BACE-1 inhibitors, and antioxidants. The optimized DE extract possessed various compounds, particularly flavonoids. To the best of our knowledge, this is the first study to report the metabolomic phytochemical profiles of DE. The extract also acted synergistically with donepezil to inhibit BACE-1 and quenched BACE-1 activities and genes downstream of amyloid aggregation in the *Drosophila* model of AD. These data suggest the promising role of DE as an anti-AD agent in vitro and in vivo. Our extraction conditions can also be used in the food, nutraceutical, and pharmaceutical industries in the future because the developed procedure was simple and accessible for a common laboratory.

## Figures and Tables

**Table 1:** BACE-1 inhibition and total phenolic contents (TPCs) of *D. esculentum* extract obtained from extraction conditions derived from BBD matrix as shown in Table 8 and Table 9.

Run	X_1_: Ethanol (% *v*/*v*)	X_2_: Time (min)	X_3_: Temperature (°C)	X_4_: SLR (g/mL)	BACE-1 Inhibition (%)		TPCs (mg GAE/g DW)
					Experimental	Predicted	Experimental
1	50	75	60	20	32.90 ± 1.38 ^F^	35.07	7.45 ± 0.07 ^JKL^
2	30	75	45	20	25.25 ± 0.40 ^GH^	17.48	7.97 ± 0.20 ^GHIJKL^
3	70	75	30	30	55.00 ± 0.97 ^ABC^	58.47	6.63 ± 0.04 ^L^
4	50	30	30	30	44.43 ± 2.33 ^DE^	40.99	8.12 ± 0.40 ^GHIJKL^
5	30	75	60	30	21.05 ± 1.14 ^HI^	22.38	10.76 ± 0.14 ^BC^
6	50	30	60	30	42.25 ± 0.74 ^E^	37.26	9.54 ± 0.66 ^CDEFG^
7	50	75	45	30	43.24 ± 1.42 ^DE^	43.95	9.48 ± 0.66 ^CDEFGH^
8	70	75	45	20	52.61 ± 0.17 ^C^	51.32	15.63 ± 1.09 ^A^
9	50	120	30	30	44.90 ± 2.18 ^DE^	43.79	8.64 ± 0.44 ^EFGHIJK^
10	70	75	60	30	57.50 ± 0.83 ^AB^	59.52	10.97 ± 0.87 ^BC^
11	30	75	45	40	25.78 ± 0.34 ^GH^	20.97	10.86 ± 0.01 ^BC^
12	70	30	45	30	57.94 ± 0.63 ^A^	54.65	10.75 ± 0.95 ^BCD^
13	50	120	45	20	29.68 ± 1.11 ^FG^	32.02	8.15 ± 0.27 ^FGHIJKL^
14	50	30	45	40	28.76 ± 1.53 ^G^	31.22	9.17 ± 0.16 ^CDEFGHIJ^
15	30	75	30	30	24.58 ± 0.64 ^H^	27.37	8.36 ± 0.06 ^FGHIJKL^
16	30	120	45	30	20.51 ± 2.13 ^I^	25.09	9.34 ± 0.41 ^CDEFGHI^
17	50	75	30	20	42.32 ± 1.71 ^DE^	41.49	7.36 ± 0.27 ^KL^
18	50	75	60	40	41.17 ± 0.51 ^E^	43.29	10.92 ± 0.31 ^BC^
19	50	120	60	30	46.24 ± 1.79 ^DE^	43.58	11.75 ± 0.98 ^B^
20	70	75	45	40	53.69 ± 1.03 ^BC^	55.36	16.40 ± 1.48 ^A^
21	50	75	30	40	41.68 ± 0.61 ^E^	40.80	8.82 ± 0.11 ^DEFGHIJK^
22	50	75	45	30	42.290 ± 2.17 ^E^	43.95	9.69 ± 0.55 ^CDEFG^
23	70	120	45	30	57.10 ± 0.79 ^AB^	54.51	7.68 ± 0.35 ^IJKL^
24	50	30	45	20	30.34 ± 1.49 ^FG^	35.71	7.79 ± 0.18 ^HIJKL^
25	30	30	45	30	11.95 ± 0.67 ^J^	15.83	9.87 ± 0.33 ^CDEF^
26	50	120	45	40	44.61 ± 0.62 ^DE^	44.04	10.12 ± 0.39 ^BCDE^
27	50	75	45	30	46.33 ± 0.65 ^D^	43.95	8.99 ± 0.13 ^DEFGHIJK^

Results are shown as mean ± standard deviation (SD) of triplicate experiments (*n* = 3). Superscript letters in each column designate significantly different BACE-1 or TPCs in each run condition derived from Table 9 at *p* < 0.05. The *p* value was determined by one-way analysis of variance (ANOVA) and Duncan’s multiple comparison test. GAE: gallic acid equivalent; DW: dry weight; SLR: solid–liquid ratio.

**Table 2:** Analysis of variance, regression coefficients, and *p* value of the second-order polynomial models for BACE-1 inhibition and TPCs derived from Table 1.

Source	BACE-1 Inhibition			TPCs		
	Coefficient	*p* Value	Significance	Coefficient	*p* Value	Significance
Model	3944.01	<0.0001	****	64.70	0.6336	ns
$X_{1}$	3492.52	<0.0001	****	9.87	0.2075	
$X_{2}$	62.43	0.1233		0.0161	0.9579	
$X_{3}$	11.60	0.4886		15.09	0.1254	
$X_{4}$	42.53	0.1964		11.89	0.1694	
$X_{1}X_{2}$	22.09	0.3436		1.62	0.5988	
$X_{1}X_{3}$	9.09	0.5390		0.9438	0.6876	
$X_{1}X_{4}$	0.0756	0.9549		1.13	0.6598	
$X_{2}X_{3}$	3.10	0.7184		0.7200	0.7252	
$X_{2}X_{4}$	68.15	0.1089		0.0876	0.9022	
$X_{3}X_{4}$	19.85	0.3685		1.01	0.6775	
$X_{1}^{2}$	46.41	0.1785		8.34	0.2443	
$X_{2}^{2}$	64.64	0.1175		1.55	0.6072	
$X_{3}^{2}$	4.64	0.6595		2.91	0.4830	
$X_{4}^{2}$	118.88	0.0411	*	2.45	0.5196	
R^2^	0.9353			0.4923		
R^2^ adjusted	0.8599			−0.1001		
Lack of fit	263.78	0.1533	ns	66.48	0.0192	*

Statistical analyses were evaluated by one-way analysis of variance (ANOVA). * *p* < 0.05; **** *p* < 0.0001; ns, not significant; X_1_, ethanol concentration; X_2_, extraction time; X_3_, extraction temperature; X_4_, solid–liquid ratio (SLR).

**Table 3:** Metabolomic profiles of the optimized *D. esculentum* extract using HPLC-qTOF/MS in positive ion full scan mode. RT, retention time.

No	RT (min)	Tentative Compounds	Mode	Molecular Mass	Precursor Mass	Formula	Library Score	Relative Area (%)
1	0.84	**Histidine**	M^+^	156.0776	156.077	C_6_N_9_H_3_O_2_	98.2	0.053
2	0.98	**Proline**	M^+^	116.0712	116.072	C_5_H_9_NO_2_	98.5	2.305
3	1	**L-Valine**	M^+^	118.0872	118.087	C_5_H_11_NO_2_	98	0.911
4	1.21	**Phenylalanine**	M^+^	166.0852	166.084	C_9_H_11_NO_2_	98.8	0.061
5	1.41	**Adenosine**	M^+^	268.1073	268.108	C_10_H_13_N_5_O_4_	100	3.407
6	1.47	**Isoleucine**	M^+^	132.1043	132.104	C_6_H_13_NO_2_	99	3.114
7	1.48	**Guanosine**	M^+^	284.0995	284.1	C_10_H_13_N_5_O_5_	100	0.115
8	2.59	**Pantothenic acid**	M^+^	220.1205	220.12	C_9_H_17_NO_5_	95	2.708
9	3.5	**Phenylethylamine**	M^+^	122.0975	122.097	C_8_H_11_N	98.9	0.565
10	3.62	**Nicotinic acid**	M^+^	124.0396	124.04	C_6_H_5_NO_2_	96.3	0.027
11	4	**5′-S-Methyl-5′-thioadenosine**	M^+^	298.0992	298.1	C_11_H_15_N_5_O_3_S	95.7	2.245
12	4.11	**L-Tryptophan**	[M^+^H]^+^	205.0987	205.099	C_11_H_11_N_2_O_2_	94.4	3.350
13	4.11	**Indole-4-carboxaldehyde**	[M^+^H]^+^	146.0608	146.061	C_9_H_7_NO	96	0.735
14	4.12	**3-Indoleacrylic acid**	M^+^	188.0741	188.075	C_11_H_9_NO_2_	93.7	11.811
15	4.83	**Tryptamine**	M^+^	161.1072	161.107	C_10_H_12_N_2_	98.1	0.048
16	5.28	**Isoferulic acid**	[M^+^CH_3_OH^+^H]^+^	195.0663	195.066	C_10_H_10_O_4_	97.7	0.157
17	5.38	**Kaempferol-3-gentiobioside**	M^+^	611.1612	611.161	C_27_H_30_O_16_	98.5	0.280
18	5.41	**2-Phenylacetamide**	[M^+^H]^+^	136.076	136.076	C_8_H_9_NO	93.9	0.012
19	5.64	**Nicotinic acid**	M^+^	124.0406	124.041	C_6_H_5_NO_2_	95.9	0.339
20	5.85	**Ritalinic acid**	M^+^	220.134	220.134	C_13_H_17_NO_2_	94.6	0.035
21	6.58	**Rutin**	[M^+^CH_3_OH^+^H]^+^	611.1673	611.166	C_27_H_36_O_19_	97.2	5.074
22	6.82	**Quercetin-3-O-galactoside**	M^+^	465.108	465.108	C_21_H_20_O_12_	100	3.409
23	6.82	**Quercetin**	[M^+^K]^+^	303.0533	303.053	C_15_H_10_O_7_	100	1.824
24	7.07	**Kaempferol-3-O-rutinoside**	[M^+^H]^+^	595.172	595.171	C_27_H_30_O_15_	97.6	4.458
25	7.34	**Kaempferol-3-O-glucoside**	M^+^	449.1148	449.114	C_21_H_20_O_11_	100	6.280
26	7.47	**Indole-6-carboxaldehyde**	M^+^	146.0609	146.061	C_9_H_7_NO	96.9	0.060
27	8.75	**Cinnamaldehyde**	M^+^	133.1014	133.066	C_9_H_8_O	92.7	0.082
28	9.08	**Bornyl acetate**	M^+^	137.1335	137.133	C_12_H_20_O_2_	97.9	1.887
29	9.1	**Niacin**	M^+^	124.0422	124.043	C_6_H_5_NO_2_	99.8	18.000
30	10.35	**Germacrone**	M^+^	219.1749	219.175	C_15_H_22_O	92.3	0.015
31	12.78	**Dinor-12-oxophytodienoic acid**	M^+^	265.1809	265.181	C_16_H_24_O_3_	93.7	0.122
32	13.29	**Piperine**	M^+^	286.1447	286.153	C_17_H_19_NO_3_	94.8	0.003
33	13.52	**Capsaicin**	M^+^	306.2068	306.211	C_18_H_27_NO_3_	95.3	0.005
34	14.4	**Monolinolenin**	M^+^	353.2695	353.269	C_21_H_36_O_4_	98.2	0.232
35	15.22	**(E)-Chalcone**	M^+^	209.0969	209.097	C_15_H_12_O	92.9	0.021
36	15.7	**Mead acid**	[M^+^Na]^+^	301.2167	301.217	C_20_H_34_O_2_	92	0.069
37	15.87	**Monolinolenin**	[M^+^CH_3_OH^+^H]^+^	353.2701	353.27	C_21_H_36_O_4_	98.2	0.759
38	16.53	**5-Hydroxy-6E,8Z,11Z,14Z-eicosatetraenoic acid**	M^+^	303.2333	303.233	C_20_H_32_O_3_	95.9	0.280
39	16.63	**15(R)-15-Methyl prostaglandin F2a methyl ester**	M^+^	365.2692	365.269	C_22_H_38_O_5_	99.4	0.055
40	17.24	**1,2-dioleoyl-sn-glycero-3-phosphatidylcholine**	M^+^	786.5974	786.598	C_44_H_84_NO_8_P	99.6	0.019
41	18.17	**1-Palmitoyl-sn-glycero-3-phosphocholine**	M^+^	496.342	496.342	C_24_H_50_NO_7_P	99.6	2.494
42	18.58	**Monolinolenin (9c,12c,15c)**	M^+^	353.2702	353.27	C_21_H_36_O_4_	98.3	0.885
43	18.71	**1-Oleoyl-sn-glycero-3-phosphocholine**	M^+^	522.3549	522.355	C_26_H_52_NO_7_P	98.1	0.081
44	18.96	**3-Isomangostin**	M^+^	411.1811	411.181	C_24_H_26_O_6_	97.1	0.218
45	19.52	**5-Hydroxy-6E,8Z,11Z,14Z-eicosatetraenoic acid, 1,5-lactone**	M^+^	303.2334	303.233	C_20_H_32_O_3_	97.2	0.466
46	19.64	**1-Arachidonoylglycerol**	[M^+^H]^+^	379.2854	379.285	C_23_H_38_O_4_	97	1.363
47	19.91	**1-Monolinolein**	[M^+^H]^+^	355.2856	355.287	C_21_H_38_O_4_	97	2.242
48	20.66	**cis-5,8,11,14-Eicosatetraenoic acid**	M^+^	305.2502	305.25	C_20_H_32_O_2_	95.2	3.326
49	23.05	**1-Stearoyl-rac-glycerol**	M^+^	359.3156	359.316	C_21_H_42_O_4_	92.9	0.185
50	23.61	**1,2-Benzenedicarboxylic acid**	M^+^	167.0341	167.034	C_6_H_8_O_4_	93.9	0.250
51	25.17	**Erucamide**	M^+^	338.3451	338.345	C_22_H_43_NO	96.8	12.847
52	26.15	**Campesterol**	M^+^	383.3663	383.366	C_28_H_48_O	90.8	0.024
53	26.3	**Monolinolenin (9c,12c,15c)**	M^+^	353.2693	353.269	C_21_H_36_O_4_	98	0.594
54	26.64	**1-Palmitoyl-2-linoleoyl-sn-glycero-3-phosphocholine**	M^+^	758.5696	758.569	C_42_H_82_NO_8_P	99.3	0.094
55	19.52	**5-Hydroxy-6E,8Z,11Z,14Z-eicosatetraenoic acid, 1,5-lactone**	M^+^	303.2334	303.233	C_20_H_32_O_3_	97.2	0.466

**Table 4:** Metabolomic profiles of the optimized *D. esculentum* extract using HPLC-qTOF/MS in negative ion full scan mode. RT, retention time.

No	RT (min)	Tentative Compounds	Mode	Molecular Mass	Precursor Mass	Formula	Library Score	Relative Area (%)
1	1.91	**2-(2-Hydroxyethoxy)phenol**	M^−^	152.9947	152.969	C_8_H_10_O_3_	93	0.0782
2	2.02	**L-Malic acid**	M^−^	133.0163	133.016	C_4_H_6_O_5_	97.5	11.6996
3	2.13	**Amber Acid**	M^−^	117.0218	117.022	C_4_H_6_O_4_	94.4	7.9976
4	2.18	**Danshensu**	[M^+^FA^−^H]^−^	197.0452	197.045	C_5_H_10_O_5_	93.6	0.4615
5	2.45	**DL-Ornithine**	[M^−^H]^−^	113.0605	113.061	C_5_H_12_N_2_O_2_	94.5	0.5874
6	2.82	**Adenine**	M^−^	134.051	134.05	C_5_H_5_N_5_	98.3	7.6981
7	4.09	**L-Tryptophan**	M^−^	203.0892	203.094	C_11_H_12_N_2_O_2_	96.6	3.7078
8	4.8	**Rosmarinic acid**	M^−^	359.0769	359.077	C_18_H_16_O_8_	99.6	0.6917
9	5.39	**3,4-Dihydroxybenzaldehyde**	M^−^	137.0257	137.025	C_7_H_8_O_3_	98	1.0651
10	6.78	**(±)-Abscisic acid**	M^−^	263.1275	263.128	C_15_H_20_O_4_	98.7	0.0844
11	6.81	**Esculetin**	M^−^	177.019	177.022	C_9_H_6_O_4_	98.2	0.2157
12	7.59	**Rutin**	M^−^	609.1579	609.193	C_27_H_36_O_19_	95.2	6.8635
13	7.8	**Quercetin-3-O-galactoside**	M^−^	463.0967	463.093	C_21_H_20_O_12_	99.4	10.0198
14	8.07	**Kaempferol-3-O-rutinoside**	M^−^	593.1646	593.164	C_27_H_30_O_15_	99.4	8.6984
15	8.39	**Kaempferol-3-O-glucoside**	M^−^	447.1005	447.101	C_21_H_20_O_11_	100	12.8390
16	10.04	**Nicotinic acid**	M^−^	122.0243	122.024	C_6_H_5_NO_2_	92.1	0.1364
17	11.65	**Liriopesides B**	[M^+^FA^−^H]^−^	767.4056	767.406	C_39_H_62_O_12_	95.3	0.2600
18	13.35	**1-Hexadecanoyl-sn-glycero-3-phospho-(1′-myo-inositol)**	M^−^	571.2884	571.295	C_25_H_49_O_12_P	90.8	1.2154
19	13.68	**Dinor-12-oxophytodienoic acid**	[M^−^H_2_O^−^H]^−^	263.1645	263.164	C_16_H_24_O_3_	93	0.7452
20	14.16	**(3.beta.)-Allopregnanolone sulfate**	M^−^	397.2245	397.225	C_21_H_33_NaO_5_S	100	0.5206
21	17.04	**1-Stearoyl-2-hydroxy-sn-glycero-3-phosphate**	M^−^	437.2648	437.265	C_21_H_43_O_7_P	96.2	0.3258
22	18.01	**9R-Hydroxy-10E,12Z-octadecadienoic acid**	M^−^	295.234	295.234	C_18_H_32_O_3_	95.4	6.3714
23	19.61	**Echinocystic acid**	M^−^	471.3461	471.346	C_30_H_48_O_4_	90.6	0.0807
24	20.03	**α** **-Mangostin**	M^−^	409.1637	409.164	C_24_H_26_O_6_	99.5	0.1506
25	21.48	**Pinolenic acid**	M^−^	277.2312	277.23	C_18_H_30_O_2_	93.3	16.5570
26	22.59	**5Z,11Z,14Z-Eicosatrienoic acid**	[M^−^H]^−^	305.2484	305.248	C_20_H_34_O_2_	98	0.6778
27	25.47	**Ginkgolic acid II**	M^−^	373.2733	373.273	C_24_H_38_O_3_	96.6	0.0982
28	28.36	**1-Palmitoyl-2-oleoyl-sn-glycero-3-phosphate**	M^−^	673.4778	673.482	C_37_H_71_O_8_P	99.9	0.1256
29	28.61	**1-Palmitoyl-2-linoleoyl-sn-glycero-3-phosphate**	M^−^	671.4612	671.467	C_37_H_69_O_8_P	98.6	0.0274

**Table 5:** Phytochemical analysis (targeted analysis) of the optimized *D. esculentum* extract.

Compounds	Amount (µg/g DW)
**Fisetin**	111.96 ± 11.89 ^C^
**Kaempferol**	342.86 ± 5.50 ^A^
**Quercetin**	133.18 ± 5.50 ^B^
**Rosmarinic acid**	52.41 ± 0.05 ^D^
**Rutin**	6.49 ± 0.10 ^E^

All data are expressed as mean ± standard deviation (SD) of triplicate experiments (*n* = 3). Different uppercase letters (A, B, C, D, and E) denote significantly different values in each column at *p* < 0.05 using one-way analysis of variance (ANOVA), followed by Duncan’s multiple comparison test.

**Table 6:** Total phenolic contents (TPCs), total flavonoid contents (TFCs), antioxidant activities (DPPH, FRAP, and ORAC), and BACE-1 inhibitory activities against of *D. esculentum* extract obtained from non-optimized and optimized extraction.

Assay	Non-Optimized	Optimized	Fold Changes (Optimized vs. Non-Optimized)
TPCs (mg GAE/g DW)	3.88 ± 0.04	13.46 ± 0.20 ***	3.47
TFCs (mg QE/g DW)	12.66 ± 0.27	30.20 ± 2.96 ***	2.39
DPPH (µmol TE/g DW)	11.15 ± 0.34	37.79 ± 0.96 ***	3.40
FRAP (µmol TE/g DW)	27.72 ± 0.29	64.12 ± 0.78 ***	2.31
ORAC (µmol TE/g DW)	93.79 ± 3.51	471.23 ± 51.99 ***	5.02
BACE-1 (% inhibition)	49.70 ± 2.97	56.33 ± 0.36 *	1.13

Data are presented as mean ± SD of three experiments (*n* = 3). Statistical analyses were determined by unpaired *t*-test. * *p* < 0.05; *** *p* < 0.001. In BACE-1 assay, DE concentration was 2 mg/mL.

**Table 7:** Synergistic effect between donepezil and optimized DE extract on BACE-1 inhibition.

Sample	BACE-1 Inhibition (%)		Interpretation
	Experimental Value (EV)	Theoretical Value (TV)	
10 µg/mL (IC_10_) DON	8.77 ± 1.06	-	-
40 µg/mL (IC_25_) DON	25.97 ± 3.49	-	-
150 µg/mL (IC_50_) DON	47.87 ± 0.99	-	-
0.125 mg/mL DE	25.92 ± 1.16	-	-
0.25 mg/mL DE	40.26 ± 3.31	-	-
0.50 mg/mL DE	47.65 ± 0.30	-	-
IC_10_ DON ± 0.125 mg/mL DE	40.41 ± 2.13	17.35	synergistic
IC_10_ DON ± 0.25 mg/mL DE	56.74 ± 3.15	24.52	synergistic
IC_10_ DON ± 0.50 mg/mL DE	40.28 ± 0.90	NA	NA
IC_25_ DON ± 0.125 mg/mL DE	51.51 ± 1.42	25.95	synergistic
IC_25_ DON ± 0.25 mg/mL DE	61.06 ± 1.39	NA	NA
IC_25_ DON ± 0.50 mg/mL DE	65.40 ± 0.83	NA	NA
IC_50_ DON ± 0.125 mg/mL DE	63.07 ± 7.46	NA	NA
IC_50_ DON ± 0.25 mg/mL DE	81.02 ± 2.33	NA	NA
IC_50_ DON ± 0.50 mg/mL DE	63.22 ± 1.99	NA	NA

Data are presented as mean ± SD of the three experiments (*n* = 3). NA = not applicable.

## Data Availability

Data are contained within this article/Supplementary Materials; further inquiries can be directed to the corresponding author.

## Supplementary Materials

The following supporting information can be downloaded at: https://www.mdpi.com/article/10.3390/molecules29102204/s1, Supplementary Table S1: Fragment ions of twenty-six phenolic standards using liquid chromatography–electrospray ionization–tandem mass spectrometry (LC-ESI-MS/MS). Supplementary Table S2: The validation parameters of twenty-six phenolic standards using liquid chromatography–electrospray ionization–tandem mass spectrometry (LC-ESI-MS/MS). Supplementary Table S3: The list and sequences of primers used in the present study. Supplementary Figure S1: Full liquid chromatography–electrospray ionization–tandem mass spectrometry (LC-ESI-MS/MS) chromatograms of the optimized *D. esculentum* extract (DE extract) presenting five detected compounds, including 1: rutin; 2: rosmarinic acid; 3: fisetin; 4: quercetin; and 5: kaempferol [56].
