# Supplementary material for: Box–Behnken Design-Based Optimization of Phytochemical Extraction from Diplazium esculentum (Retz.) Sw. Associated with Its Antioxidant and Anti-Alzheimer’s Properties
Source: Molecules. 2024 May 8;29(10):2204. doi: 10.3390/molecules29102204 (PMC11124457; doi:10.3390/molecules29102204)

molecules

Supplementary Materials

Box–Behnken Design-Based Optimization of Phytochemical Extraction from *Diplazium esculentum* (Retz.) Sw. Associated with Its Antioxidant and Anti-Alzheimer's Properties

Sirawit Kongsung [1], Woorawee Inthachat [2], Boonrat Chantong [3], Uthaiwan Suttisansanee [2], Nattira On-Nom [2], Chaowanee Chupeerach [2], Sirinapa Thangsiri [2], Pornsiri Pitchakarn [4,*] and Piya Temviriyanukul [1,*]

[1] Master of Science Program in Toxicology and Nutrition for Food Safety, Institute of Nutrition, Mahidol University, Nakhon Pathom 73170, Thailand; sirawit.kon@student.mahidol.ac.th (S.K.)
[2] Institute of Nutrition, Mahidol University, Salaya, Phuttamonthon, Nakhon Pathom 73170, Thailand; woorawee.int@mahidol.ac.th (W.I.); uthaiwan.sut@mahidol.ac.th (U.S.); nattira.onn@mahidol.ac.th (N.O.); chaowanee.chu@mahidol.ac.th (C.C.); sirinapa.tha@mahidol.ac.th (S.T.)
[3] Department of Pre-clinical and Applied Animal Science, Faculty of Veterinary Science, Mahidol University, Salaya, Phutthamonthon, Nakhon Pathom 73170, Thailand.; boonrat.cha@mahidol.ac.th (B.C.)
[4] Department of Biochemistry, Faculty of Medicine, Chiang Mai University, Chiang Mai 50200, Thailand.; pornsiri.p@cmu.ac.th (P.P.)
* Correspondence: pornsiri.p@cmu.ac.th (P.P.) and piya.tem@mahidol.ac.th (P.T.)

Supplementary Table S1:

Fragment ions of twenty-six phenolic standards using liquid chromatography–electrospray ionization–tandem mass spectrometry (LC-ESI-MS/MS). The validation data were obtained from our previus works (Sirichai et.al., 2022 and Koirala et al., 2024).

Compounds	Standards	Ion mass	Parent ions (*m*/*z*)	SRM transitions (*m*/*z*) and collision energy (*V*)	*RF lens (V)*
1	Epigallocatechin gallate	[M–H]	457.175	305.155 (16.84 V), 168.97 (17.59 V), 125.042 (40.30 V)	204
2	Gallic acid	[M–H]	169.05	124.988 (14.56 V), 96.917 (18.77 V), 79.185 (22.94 V)	147
3	3,4-Dihydroxybenzoic acid	[M–H]	152.95	109.113 (14.35 V), 81.042 (20.50 V), 91.042 (24.59 V)	128
4	Chlorogenic acid	[M–H]	353.075	179.042 (14.06 V), 191.000 (16.54 V), 85.095 (39.96 V)	148
5	4-Hydroxybenic acid	[M–H]	137.05	92.970 (14.86 V), 65.000 (29.39 V), 75.000 (31.96 V)	110
6	Caffeic acid	[M–H]	179.038	135.054 (15.07 V), 107.071 (22.57 V), 85.042 (31.96 V)	151
7	Syringic acid	[M–H]	197.138	182.185 (13.72 V), 167.113 (19.24 V), 123.095 (22.31 V)	130
8	Vanillic acid	[M–H]	167.000	123.042 (11.66 V), 151.97 (14.59 V), 108.042 (18.65 V)	114
9	*p*-Coumaric acid	[M+H]	165.05	147.054 (11.70 V), 119.113 (19.36 V), 91.125 (25.89 V)	90
10	Rutin	[M+H]	611.20	303.13 (20.80), 465.20 (12.71V)	198
11	Sinapic acid	[M–H]	223.25	208.125 (13.51 V), 164.024 (15.78 V), 192.970 (22.65 V)	141
12	Ferulic acid	[M–H]	192.95	149.125 (11.28 V), 177.970 (13.05 V), 134.042 (16.50 V)	124
13	Hesperidin	[M–H]	609.30	301.179 (24.50 V), 325.179 (27.83 V), 286.125 (41.60 V)	299
14	Myricetin	[M–H]	317.088	178.970 (19.53 V), 150.988 (24.50 V), 137.113 (26.86 V)	245
15	Rosmarinic acid	[M–H]	359.20	197.000 (15.70 V), 161.113 (17.38 V), 133.054 (37.81 V)	175
16	Luteolin	[M–H]	285.138	197.000 (15.70 V), 161.113 (17.38 V), 133.054 (37.81 V)	241
17	Quercetin	[M–H]	301.200	178.976 (18.18 V), 273.125 (19.45 V), 151.042 (21.39 V)	237
18	Cinnamic acid	[M–H]	147.00	103.00 (11.23V), 77.083 (23.07)	107
19	Apigenin	[M–H]	269.075	116.863 (34.28 V), 149.071 (25.13 V), 151.131 (25.05 V)	244
20	Genistein	[M–H]	269.138	224.054 (25.60 V), 159.054 (29.26 V), 132.929 (30.95 V)	239
21	Naringenin	[M+H]	272.938	146.97 (21.01 V), 153.054 (24.42 V), 119.000 (31.28 V)	160

Supplementary Table S1 (Cont.):

Fragment ions of twenty-six phenolic standards using liquid chromatography–electrospray ionization–tandem mass spectrometry (LC-ESI-MS/MS). The validation data were obtained from our previus works (Sirichai et.al., 2022 and Koirala et al., 2024).

Compounds	Standards	Ion mass	Parent ions (*m*/*z*)	*SRM* transitions (*m*/*z*) and collision energy (*V*)	*RF lens (V)*
22	Kaempferol	[M–H]	285.150	184.911 (25.85 V), 239.113 (27.03 V), 186.988 (28.17 V)	260
23	Isorhamnetin	[M–H]	315.088	300.000 (21.30 V), 150.970 (29.14 V), 271.054 (30.57 V)	233
24	Galangin	[M+H]	271.088	165.042 (28.80 V), 197.125 (31.75 V), 153.113 (32.42 V)	248
25	Fisetin	[M+H]	287.050	213.054 (27.96V), 137.042 (30.86V), 241.125 (25.09V)	244
26	Mangiferin	[M+H]	423.100	273.054 (23.58V), 303.113 (17.7V), 327.071 (16.88V)	108

References:

Sirichai, P.; Kittibunchakul, S.; Thangsiri, S.; On-Nom, N.; Chupeerach, C.; Temviriyanukul, P.; Inthachat, W.; Nuchuchua, O.; Aursalung, A.; Sahasakul, Y.; et al. Impact of Drying Processes on Phenolics and In Vitro Health-Related Activities of Indigenous Plants in Thailand. Plants 2022, 11, 294. https://doi.org/10.3390/plants11030294.

Koirala P, Chunhavacharatorn P, Suttisansanee U, Benjakul S, Katewongsa K, Al-Asmari F, Nirmal N. Antioxidant and antimicrobial activities of mango peel and radish peel-a comparative investigation. Front. Sustain. Food Syst. 2024; 8:1354393. doi: 10.3389/fsufs.2024.1354393.

Supplementary Table S2:

The validation parameters of twenty-six phenolic standards using liquid chromatography–electrospray ionization–tandem mass spectrometry (LC-ESI-MS/MS) .The validation data were obtained from our previus work (Sirichai et.al., 2022 and Koirala et al., 2024).

Compounds	Retention time (min)	Standards	Linear range (µg/mL)	Linear regression equation	Correlation coefficient (R^2)	LOD (µg/mL)	LOQ (µg/mL)	%RSD (Inter-day)	%Recovery		
									Low level (µg/mL)	Medium level (µg/mL)	High level (µg/mL)
1	0.44	Epigallocatechin gallate	0.125–40	y =8533x + 1053.4	0.9985	0.067	0.230	0.023	91.84	85.36	91.37
2	0.564	Gallic acid	0.195–25	y =3323.1x – 2100.4	0.9984	0.04	0.14	0.01	113.05	118.57	109.12
3	0.803	3,4-Dihydroxybenzoic acid	0.195–25	y =11490x – 10877	0.9935	0.010	0.034	0.003	90.59	85.75	89.75
4	0.922	Chlorogenic acid	0.3125–40	y =8377.5x – 3623.5	0.9934	0.017	0.055	0.006	91.94	87.50	95.02
5	1.16	4-Hydroxybenic acid	0.3125–40	y =2482.6x– 3998.4	0.9917	0.027	0.090	0.009	109.67	103.60	101.28
6	1.40	Caffeic acid	0.3125–40	y =12328x–19725	0.9918	0.010	0.035	0.003	105.36	93.98	87.41
7	1.539	Syringic acid	3.125–100	y =68.091x + 230.43	0.9955	0.582	1.939	0.194	116.35	97.42	94.91
8	1.63	Vanillic acid	2.5–100	y =213.67x – 975.72	0.9900	0.15	0.48	0.05	99.86	101.76	100.12
9	2.452	*p*-Coumaric acid	0.3125–40	y =8532.4x–13559	0.9910	0.013	0.042	0.004	88.22	81.36	98.05
10	2.737	Rutin	0.009–1.25	y =49729x – 33.064	0.9999	0.001	0.005	0.0005	94.63	114.00	108.73
11	2.772	Sinapic acid	0.39–25	y =1592.6x – 832.22	0.9977	0.026	0.086	0.009	81.34	92.16	84.22
12	2.851	Ferulic acid	1.56–100	y =559.03x – 1819.2	0.9947	0.155	0.518	0.052	91.51	89.24	93.10
13	3.41	Hesperidin	0.25–40	y =838.63x – 242.2	0.9986	0.07	0.22	0.02	100.43	104.06	108.60
14	3.431	Myricetin	1.25–40	y =303.47x – 601.81	0.9976	0.261	0.871	0.087	113.07	81.77	91.12
15	3.528	Rosmarinic acid	0.3125–40	y =4322.4x-3744.1	0.9956	0.07	0.25	0.02	92.45	106.35	99.62
16	4.158	Luteolin	0.195–12.5	y =8381.9x – 5000.7	0.9945	0.015	0.050	0.0005	84.21	96.21	107.09
17	4.185	Quercetin	0.05–12.5	y =2934x + 917.17	0.9937	0.05	0.18	0.02	83.36	115.06	95.74

Supplementary Table S2 (Cont.):

The validation parameters of twenty-six phenolic standards using liquid chromatography–electrospray ionization–tandem mass spectrometry (LC-ESI-MS/MS) .The validation data were obtained from our previus work (Sirichai et.al., 2022 and Koirala et al., 2024).

Compounds	Retention time (min)	Standards	Linear range (µg/mL)	Linear regression equation	Correlation coefficient (R^2)	LOD (µg/mL)	LOQ (µg/mL)	%RSD (Inter-day)	%Recovery		
									Low level (µg/mL)	Medium level (µg/mL)	High level (µg/mL)
18	4.522	Cinnamic acid	0.039–10	y =6631.9x – 866.59	0.9964	0.049	0.163	0.016	101.94	98.84	95.85
19	4.689	Apigenin	0.34–11	y =1790.7x – 287.7	0.9997	0.127	0.424	0.042	88.84	106.89	114.79
20	4.693	Genistein	0.625–40	y =1247.2x – 1747.1	0.9977	0.049	0.163	0.016	95.33	101.49	11633
21	4.705	Naringenin	0.0008–5	y =16755x + 443.03	0.9932	0.003	0.011	0.001	117.92	96.26	111.08
22	4.79	Kaempferol	0.25–10	y =1006.8x – 346.28	0.9905	0.122	0.406	0.041	92.35	107.69	102.17
23	4.878	Isorhamnetin	0.0098–2.5	y =12698x + 586.16	0.9945	0.016	0.052	0.005	113.57	105.88	111.14
24	6.146	Galangin	0.3125–40	y =5012.1x – 9354.7	0.9879	0.010	0.035	0.003	84.01	112.92	115.80
25	3.620	Fisetin	0.25-10	y =7468.4x – 701.27	0.9979	0.150	0.490	0.05	98.01	108.94	117.51
26	1.116	Mangiferin	0.125-10	y =7516.2x + 195.48	0.9985	0.040	0.130	0.01	102.20	91.14	91.57

References:

Sirichai, P.; Kittibunchakul, S.; Thangsiri, S.; On-Nom, N.; Chupeerach, C.; Temviriyanukul, P.; Inthachat, W.; Nuchuchua, O.; Aursalung, A.; Sahasakul, Y.; et al. Impact of Drying Processes on Phenolics and In Vitro Health-Related Activities of Indigenous Plants in Thailand. Plants 2022, 11, 294. https://doi.org/10.3390/plants11030294.

Koirala P, Chunhavacharatorn P, Suttisansanee U, Benjakul S, Katewongsa K, Al-Asmari F, Nirmal N. Antioxidant and antimicrobial activities of mango peel and radish peel-a comparative investigation. Front. Sustain. Food Syst. 2024; 8:1354393. doi: 10.3389/fsufs.2024.1354393.

Supplementary Table S3: The list and sequences of primers used in the present study.

Drosophila Genes	Primers	Sequences
dmATF-6	forward	TCCTTCAGCGTGTTCTGGTG
	reverse	TGGATAGACCGCCTCTTCGT
dmBip	forward	ACCATCCCGAGGCATCAATC
	reverse	GGTCAGTGGGTTCACATCGAG
dmGPx	forward	GGCCTGGTGATCCTCAACTTC
	reverse	GATGTCAGCCTTGGAGTCGC
dmGSTD1	forward	CTGAAGCCGGAGTTCCTGAAG
	reverse	GGTCTTGCCGTACTTCTCCAC
dmIRE-1	forward	CAATGCTCCCTGTCGTTCCTC
	reverse	CTAGATAGATGGCCCGCGAAG
dmNCT	forward	GCCCACCATCTCCACCTTAC
	reverse	GTTCTTTGGTCCGGCTTCCT
dmNEP-1	forward	ATCGCCTTTACCGTTCTGGG
	reverse	TGTCATCGTTGTCTGGCTCC
dmPEK or PERK	forward	GGCACCCAACTACTGATTCG
	reverse	CGACGCTCCTCCTGATCTAC
dmRpL32	forward	AGATCGTGAAGAAGCGCACC
	reverse	CGATCCGTAACCGATGTTGG
dmSOD1	forward	CAATCCGTATGGCAAGGAGC
	reverse	CGCCGAAGAGCGTAATCTTG

Supplementary Figure S1:

Full liquid chromatography–electrospray ionization–tandem mass spectrometry (LC-ESI-MS/MS) chromatograms of the optimized D. esculentum extract (DE extract) presenting five detected compounds, including 1: rutin; 2: rosmarinic acid; 3: fisetin; 4: quercetin; and 5: kaempferol.

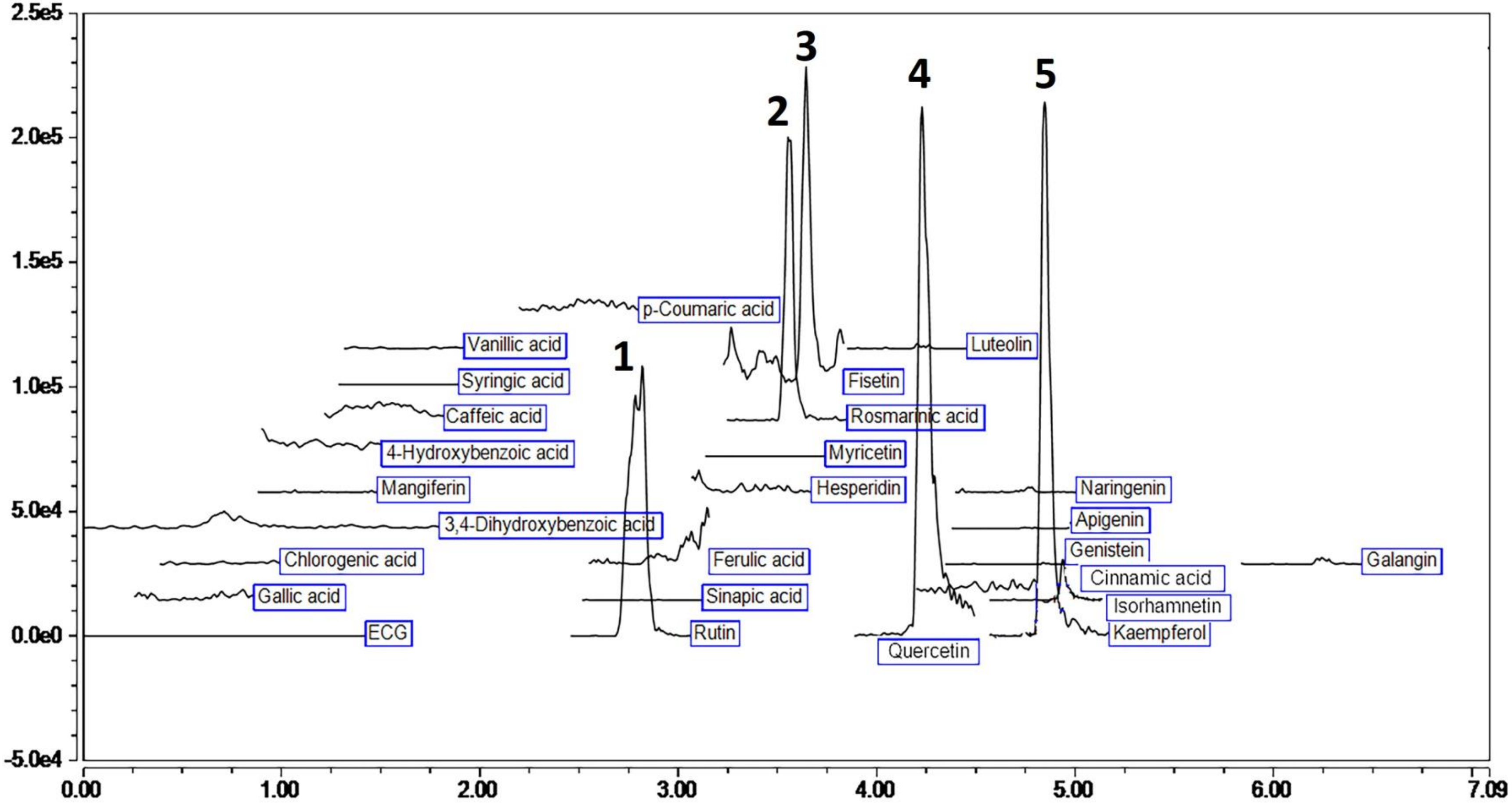

Supplement: Supplementary file 1 [file molecules-29-02204-s001.zip › molecules-2986970-supplementary.pdf]
